# PW02-024-B - First report of AA amyloidosis in Blau syndrome

**DOI:** 10.1186/1546-0096-11-S1-A165

**Published:** 2013-11-08

**Authors:** E Ruiz-Ortiz, A Saurina, E González-Roca, M Solé, C Modesto, F Rius, S Plaza, J Yagüe, JI Aróstegui

**Affiliations:** 1 Immunology, Hospital Clinic, Barcelona, Spain; 2 Nephrology, Consorci Sanitari de Terrassa, Terrassa, Spain; 3 Pathology, Hospital Clinic, Barcelona, Spain; 4 Pediatric Rheumatology, Hospital Vall Hebrón, Barcelona, Spain

## Introduction

Systemic AA amyloidosis is a life-threatening complication of different chronic infectious and inflammatory diseases. The deposition of amyloid fibrils derived from the serum amyloid A (SAA) protein represents its pathological hallmark. A long lasting and increased serum level of SAA is a prerequisite to its development. The group of inherited autoinflammatory diseases includes different disorders consequence of a genetically-determined dysregulation of innate immune system. All these diseases are associated with a marked acute phase response. The incidence of AA amyloidosis varies widely among them, with the higher incidence in Muckle-Wells syndrome and in TNF receptor-associated periodic syndrome. Inversely, no cases of AA amyloidosis have been reported in some few inherited autoinflammatory diseases, including Blau syndrome, a dominantly-inherited disease caused by *NOD2* mutations.

## Case report

The patient is a 30 years-old woman that referred since the 21 months of age persistent fever, chronic polyarthritis, skin rash, and ocular manifestations including recurrent bilateral uveitis. Granulomatous infiltration was detected in synovial biopsies, with negative results for microbiologic test. Her mother and sister were also affected by a similar disease, suggesting an autosomal dominant inheritance pattern. A clinical diagnosis of Blau syndrome was proposed. The *NOD2* analyses revealed a heterozygous c.1759C>T transition that provokes the novel p.Arg587Cys mutation. This mutation was subsequently detected in her affected relatives, and established the definitive diagnosis of Blau syndrome.

At 25 year-old she became pregnant, and during the pregnancy, proteinuria was detected in the first and second quarter, without other accompanying signs. Proteinuria disappeared in the third quarter. After delivery, several episodes of intense uveitis were detected, and treated with corticosteroids. Methotrexate (MTX) was also added to modify the inflammatory disease activity and to prevent the recurrence of uveitis. However, one year later, MTX was discontinued to avoid potential teratogenic effects in a future pregnancy.

During the follow-up, different routine tests revealed repeatedly glomerular-range proteinuria, with normal urine sediment and preserved renal function. A renal biopsy was performed and pathological studies revealed Congo-red positive deposits with glomerular and vascular involvement. Immunohistochemical studies revealed that these deposits were of AA-type. Antiproteinuric measures were started, and proteinuria decreased after 5 months of treatment.

## Discussion

This case represents the first description of AA amyloidosis in Blau syndrome. It highlights the relevance of monitoring the renal function in the follow-up of patients affected by any inherited autoinflammatory disease, independently of the previously reported risk of AA amyloidosis development.

## Disclosure of interest

None declared.

